# Comparing Echo-Detection and Echo-Localization in Sighted Individuals

**DOI:** 10.1177/03010066211000617

**Published:** 2021-03-05

**Authors:** Carlos Tirado, Billy Gerdfeldter, Stina C. Kärnekull, Mats E. Nilsson

**Affiliations:** 7675 Stockholm University, Sweden

**Keywords:** detection, localization, human echolocation, Echobot

## Abstract

Echolocation is the ability to gather information from sound reflections. Most previous studies have focused on the ability to detect sound reflections, others on the ability to localize sound reflections, but no previous study has compared the two abilities in the same individuals. Our study compared echo-detection (reflecting object present or not?) and echo-localization (reflecting object to the left or right?) in 10 inexperienced sighted participants across 10 distances (1–4.25 m) to the reflecting object, using an automated system for studying human echolocation. There were substantial individual differences, particularly in the performance on the echo-localization task. However, most participants performed better on the detection than the localization task, in particular at the closest distances (1 and 1.7 m), illustrating that it sometimes may be hard to perceive whether an audible reflection came from the left or right.

Echolocation is the ability to use sound reflections to gather information about the surrounding environment (Kolarik et al., 2014; Stroffregen & Pittenger, 1995). This ability is not limited to animals such as bats or dolphins; some humans can use echolocation to navigate their environment by detecting or localizing objects (Rice, 1967, 1969; Supa et al., 1944). Human echolocation seems to involve at least two tasks, namely echo-detection (*was the object there?*) and echo-localization (*where was the object?*) of sound-reflecting objects. The purpose of the present experiment was to explore how performance on these two tasks are related, a question not addressed by previous research on human echolocation.

Most echolocation studies on humans have been performed using echo-detection tasks, that is, with focus on the participant’s ability to detect objects using echoes (Ammons et al., 1953; Cotzin & Dallenbach, 1950; Dufour et al., 2005; Kellogg, 1962; Nilsson & Schenkman, 2016; Rice et al., 1965; Schenkman & Jansson, 1986; Schenkman & Nilsson, 2010, 2011; Schörnich et al., 2012; Teng et al., 2012; Tirado et al., 2019; Tonelli et al., 2016). Studies on echo-detection typically have involved participants being seated in front of an object that would be present in some trials and absent in others. The types of objects, distances, and tools used to move the objects vary substantially between studies. Typically, participants would make their own signals, or wait for a loudspeaker to emit a click and then be asked whether the object reflected the click or not. Alternatively, participants would walk while attempting to avoid obstacles by producing clicks, or report when they felt that they were away from an obstacle (see Dodsworth et al., 2020; Kellogg, 1962; Rice, 1967; Tirado et al., 2019 for examples of different echo-detection experiments). In general, results have shown that most people are above chance level at detecting objects of different sizes and material, that signal duration improves detection performance, and that self-generated signals require training to produce efficient palatal clicks (Rice et al., 1965; Schenkman & Nilsson, 2010; Tirado et al., 2019). Furthermore, studies have shown that naïve participants can quickly learn to detect objects using echolocation (Norman & Thaler, 2018; Schenkman & Nilsson, 2011).

Fewer studies on human echolocation have been performed using echo-localization tasks, that is, with focus on the participant’s ability to localize objects using echoes (Després et al., 2005; Dufour et al., 2005; Rice, 1969; Rowan et al., 2013, 2015, 2017; Schenkman & Jansson, 1986; Teng et al., 2012; Teng & Whitney, 2011). In studies of echo-localization, participants are typically asked for the exact position of the object (e.g., Rice, 1969; Schenkman & Jansson, 1986). Another common way to measure localization ability is to ask whether an object is at their left or at their right (e.g., Després et al., 2005; Dufour et al., 2005; Rowan et al., 2013, 2015, 2017; Teng et al., 2012; Teng & Whitney, 2011). In both types of experiments, most participants can localize the objects better than chance. The size of and the distance to the reflecting object are the main difficulty parameters (the farther away and the smaller the object is, the more difficult the task), but the degree of spatial acuity varies a lot across individuals (Dufour et al., 2005; Teng et al., 2012; Teng & Whitney, 2011).

Often, detection and localization go together, that is, if one can hear a sound, one also knows which direction it comes from. However, in some situations, a clearly audible sound may be hard to localize, for example, sinusoidal signals are typically hard to localize (Yost, 1981). Another example is the precedence-effect phenomenon *discrimination suppression* (Brown & Christopher Stecker, 2013), which may be relevant for human echolocation (Nilsson et al., 2019; Nilsson & Schenkman, 2016; Wallmeier et al., 2013). In a typical discrimination suppression experiment, a lead-click from straight ahead is followed by a lag-click from the left or right. The two clicks have the same amplitude and are presented at interclick intervals (ICIs) < 10 ms. In this setup, the lag-click is clearly audible, however not as a separate event (echo) but rather as the coloration of a single click, the perceptually fused lead–lag-click. Many listeners find it hard to determine whether the lag-click came from the left or right, so this is another example of an audible sound being hard to localize. At the ear of the echolocator, the time interval between a self-generated mouth click and its reflection is roughly 6 ms per meter from the reflecting surface (Tirado et al., 2019); for distances up to about 1.5 m, the ICI (also known as echo-delay) would thus be in the range where discrimination-suppression research has found a loss of spatial information in audible clicks.

In the present experiment, we tested 10 naïve-sighted participants on their echo-detection and echo-localization abilities. An automated method, referred to as the Echobot, was used to allow rigorous psychophysical testing with real sounds, distances, and reflecting objects (Tirado et al., 2019). The purpose was to explore at what distances naïve listeners would be able to detect and localize sound reflections. Specifically, to study to what extent echo-detection also entails echo-localization, that is, whether detection of a reflection also meant that it would be possible to determine whether it came from the left or the right.

## Methods

### Materials

**Figure 1.**
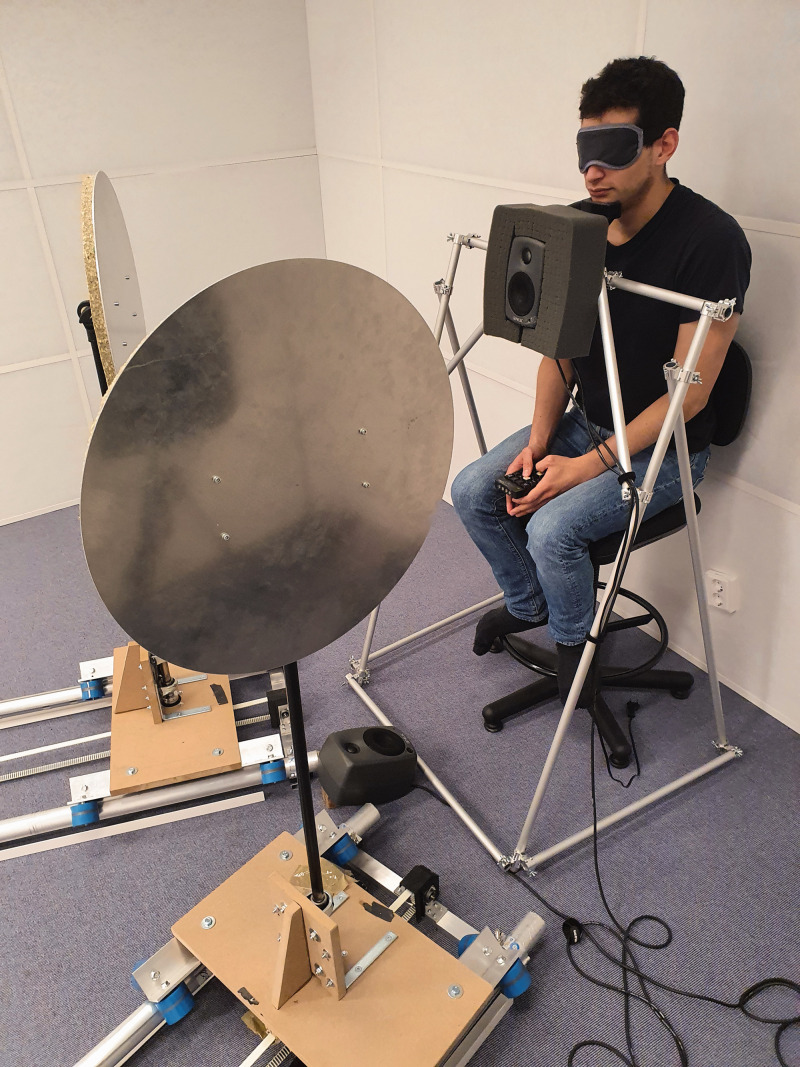
The Echobot. Blindfolded participant (first author, CT in the picture) sitting with his chin fixed behind the loudspeaker and in front of the Echobot. The loudspeaker was located 1 m away from the Echobot at a height of 1.20 m. The masking noise was played from the loudspeaker on the floor in front of the participant. The participant gave his or her responses using a small wireless numerical keyboard. Notice that the distance between the listener and reflective disks varied throughout the experiment; the distance to the disk shown in the figure is only 1 of 10 distances included in the experiment.

**Figure 2.**
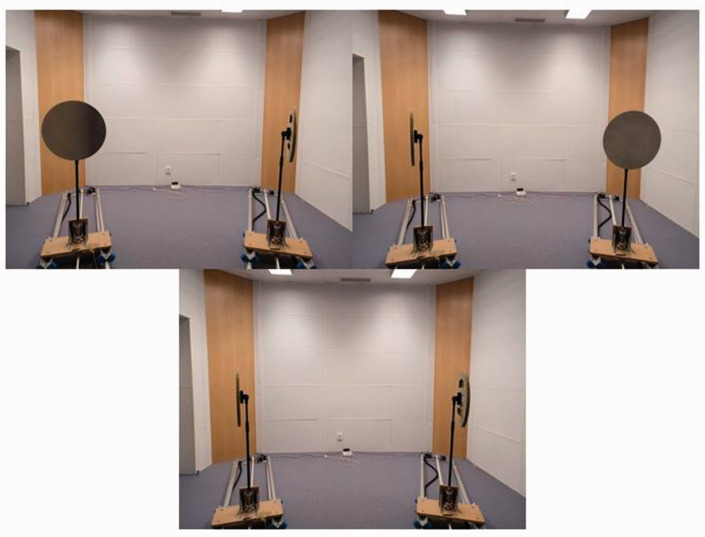
Possible positions of the Echobot. Participant’s perspective of the two disks in the three different positions used in the experiment. Top left: left disk in reflecting position (“left-reflector”). Top right: right disk in reflecting position (“right-reflector”). Lower center: both disks in nonreflecting position (“no-reflector”). The no-reflector position was only used in the echo-detection task.

An automated system, the Echobot, was used to generate stimuli for both tasks (see Figures 1 and 2). The Echobot consists of platforms that can be programmed to move across a 4 m-long rail and to horizontally rotate vertical disks of different sizes and materials. In the present experiment, it is a circular aluminum disk 50 cm in diameter and 0.4 cm thick (see Tirado et al., 2019 for further details on the Echobot). The Echobot allows for a more rigorous and time-efficient application of psychophysical methods than previous nonautomated paradigms. In the present experiment, two disks were needed to make the localization task possible (see Figure 1). They were each placed 18° laterally from the location of the participant. A loudspeaker generated a noise while the Echobot was moving to mask the sound made by its movements. The masking noise was a mix of several recordings of the Echobot. The Echobot was placed in a soundproof listening laboratory with a low background level (<20 dB[A]) and short reverberation time (<0.1 between 0.25 and 8 kHz). The floor area was 5.3 × 4.0 m^2^ with a ceiling height of 3 m (see Tirado et al., 2019 for more details on the acoustics of the masking noise and the soundproof laboratory). There was a loudspeaker rig intended for other types of experiments present in the room, but the rig was covered with soft dampening material to eliminate reflections and resonances (the rig is not visible in the photos of Figures 1 and 2). The experiment was programmed and run in Python 2.8, using the PsychoPy library (Peirce, 2007).

### Echolocation Signal

The most common sound used by human echolocators is a self-generated mouth-click, such as hissing noises or palatal clicks (see Rojas et al., 2009 for an acoustical characterization of the clicks). However, there is also evidence that echolocation using sounds present in the natural environment (Ashmead & Wall, 1999) and that signals produced by loudspeakers are as efficient as using self-generated signals (Thaler & Castillo-Serrano, 2016). In the present experiment, we used an echo signal based on simulated recordings of mouth-clicks generated by an experienced echolocator. The click was about 3 ms in duration, and dominant frequencies were around 3 to 4 kHz (see Thaler et al., 2017 for a detailed acoustic characterization of the click called EE1). The peak-to-peak sound pressure level (SPL) of the click was set to 100 dB, and the maximum SPL (1-ms time-window) was 88 dB, as measured from 1 m directly in front of the loudspeaker (Genelec Model 8010 A).

### Echo-Detection Versus Echo-Localization Experiment

The participants were seated in front of the Echobot (see Figure 1) and responded using a wireless numerical keyboard connected to the computer controlling the Echobot. The participants were blindfolded during testing to eliminate visual cues, and their chin was placed in a chin rest to eliminate head movements. A constant stimulus paradigm was used in this experiment, with the different stimuli (distances to the disk) presented in random orders. This means that the time it took the Echobot to move from one distance to the next varied between trials. Ten distances were selected in this experiment to cover the full length of the rails: 1.00, 1.36, 1.72, 2.08, 2.44, 2.81, 3.17, 3.53, 3.89, and 4.25 m from the ears of the participant.

Participants were tested during 8 days; 4 days on the echo-detection task and 4 days on the echo-localization task. Participants #2, #3, #4, #6, and #7 started with the detection task followed by the localization tasks and vice versa for Participants #1, #5, #8, #9, and #10. Each day consisted of 10 sessions. Each session contained 50 trials comprising a random order of the 10 distances repeated five times each. Participants took a short break of 1−2 minutes after every session and a longer 15-minute break after the fifth session. One day of practice seems reasonable for echolocation tasks, and it has been applied by other echolocation researchers (Maezawa & Kawahara, 2019). Therefore, the first day was treated as practice, and data analysis was based on responses from day 2 to 4 (no substantial performance differences between these days were found; see supplementary analysis, Tirado et al., 2021), in total 150 trials per distance, task, and participant.

In each trial, the click was presented once, and the trial would last until the participant responded. In the detection task, participants were asked if the disk was reflecting or not, by pressing the 8/up-key for *yes, the disk is reflecting* and the 2/down-key for *no, the disk is not reflecting* on the numerical keyboard. In the echo-localization task, participants were asked whether the reflection was coming from the left or the right (participants were aware that there was always a reflection present), by pressing the 4/right-key for *the reflection comes from the right* and the 6/left-key for *the reflection comes from the left.* In the detection task, the disks of the Echobot had three possible positions: (a) the left disk facing the participant (“left-reflector”), (b) the right disk facing the participant (“right-reflector”), and (c) no disk facing the participant (“no-reflector”), whereas in the localization task, only two of these positions were used: (a) left-reflector or (b) right-reflector (see Figure 2). In the detection task, it was randomized whether a trial would use the left-reflector position (with probability *p* = 0.25), the right-reflector position (with *p* = 0.25), or the no-reflector position (with *p* = 0.5). In the localization task, it was randomized whether a trial would use the left-reflector (with *p* = 0.5) or the right-reflector position (with *p* = 0.5).

### Participants

Ten participants were tested. They were students or staff at the Department of Psychology, Stockholm University, of which three were authors of this study (#4, #7, and #8 were C. T., B. G., and M. E. N., respectively). Participants #1, #2, #5, #6, and #10 were between 20 and 30 years old, #3, #4, #7, #8, and #9 between 30 and 40 years old, and 1 participant was between 50 and 60 years old. Six participants were female and four were male. All participants provided written informed consent before participating. An audiometer (Interacoustic Diagnostic Audiometer, model AD226) was used to measure pure-tone thresholds for the frequencies 500, 1000, 2000, 3000, 4000, and 6000 Hz (Hughson Westlake method), separately for each ear. All participants had hearing levels ≤25 dB HL in their best ear at each tested frequency. The average thresholds at the frequencies 500, 1000, 2000, and 4000 Hz (PTA4F, e.g., Nilsson & Schenkman, 2016) ranged between 1 and 6 dB HL. The study was approved by the Regional Ethics Review Board in Stockholm (214 2017/170–31/1).

### Acoustic Measurements

Acoustic measurements of the Echobot were conducted using an artificial head (Brüel & Kjær Type 4100, with microphone type 4190, preamplifier type 2669) together with an amplifier (Brüel & Kjær Type 2690 NEXUS) and an external soundcard (RME HDSPe, 48 kHz sampling frequency, 24-bit depth) connected to a computer. The artificial head was placed in the same location as the participant’s head in the experiments, just behind and above the loudspeaker that generated the echolocation click. Measurements were taken in steps of 0.05 m from 1.0 to 4.25 m distance to the reflecting disk. At each distance, measurements of the echolocation signal were taken with the Echobot in left-reflector, right-reflector, and no-reflector position.

**Figure 3.**
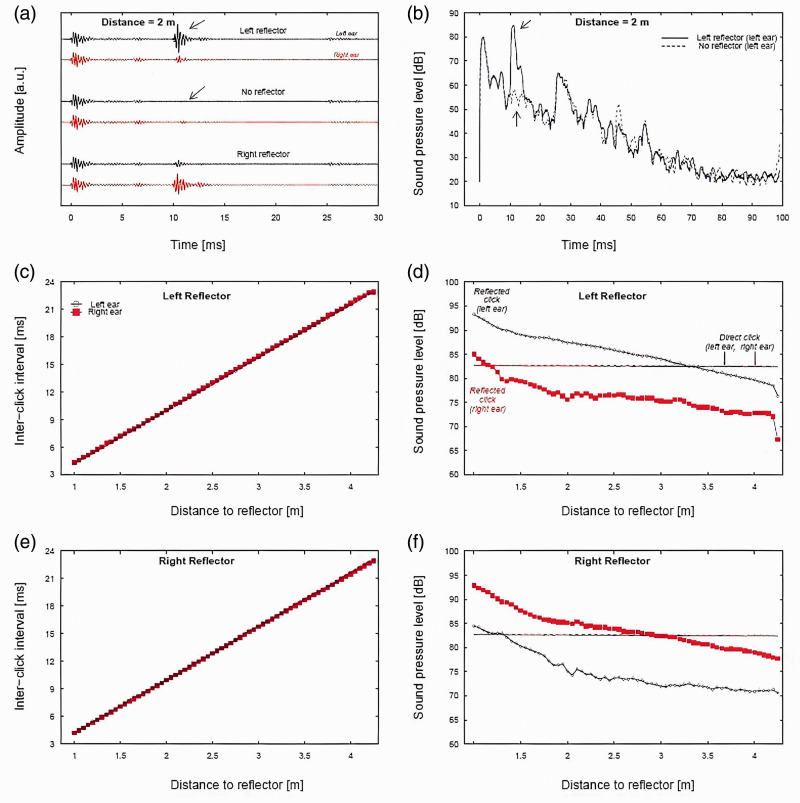
Acoustic analysis of direct and reflected clicks. Panel A: Waveforms of signals recorded with the Echobot’s disks at 2 m distance in left-reflector (upper two waveforms), no-reflector (middle two waveforms), and right-reflector position (lower two waveforms). The black arrow indicates the reflected click in the left-channel of the left-reflector measurement; the blue arrow indicates the absence of a reflected click in the left-channel of the no-reflector measurement. Panel B: Sound pressure levels (SPLs), calculated for a running and overlapping 1-ms window, as a function of time. The lines show SPLs with the Echobot in left-reflector position (solid) and in no-reflector position (dashed line). The arrows show the same component of the signals as indicated by the arrows in Panel A. Panel C shows estimates of the interclick interval (ICI) between the direct and reflected click, separately for the left (open circles) and right ear (filled squares). Panel D shows maximum SPL (1-ms rectangular window) of the reflected click as a function of distance to the reflector with the Echobot in left-reflector position, for the left (open circles) and right ear (filled squares), the horizontal lines indicate the maximum level of the direct click. Panel E corresponds to Panel C but with the Echobot in right-reflector position. Panel F corresponds to Panel D but with the Echobot in right-reflector position.

Figure 3 visualizes the binaural signals recorded from the Echobot. Note that these results refer to one set of measurements. These measurements should be interpreted as approximations of the acoustical properties of the experimental stimuli, as the results would vary slightly from recording to recording, depending on the exact positioning of the artificial head.

Panel A of Figure 3 shows waveforms from the left (black lines) and right ear (red lines), with the Echobot’s disks positioned at a 2 m distance from the ears of the artificial head in left-reflector position (upper two waveforms), no-reflector position (middle two waveforms), and in right-reflector position (lower two waveforms). The direct click is seen at 0 ms in all waveforms. The reflected click is clearly visible at around 10 ms in the upper (left-reflector) and lower (right-reflector) pairs of waveforms and is most prominent in the ear closer to the disk. As expected, there is no visible disk reflection in the middle pair of waveforms (no-reflector). Note that the amplitude of the reflected click was greater than that of the direct click at the closest distances. This is due to the directivity of the sound source relative to the receiver. The loudspeaker radiates less energy backward than forward, especially for high-frequency sounds, such as our clicks that contain dominant frequencies around 3 to 4 kHz. The receiver (microphone or ears of an echolocator in the experiment) was located behind the loudspeaker, so less sound would reach the receiver than would be transmitted forward. In contrast, the reflecting disk was located in front of the receiver and reflected most of the sound back to the receiver (the dominant frequencies of the click corresponded to wavelengths <0.12 m, which would be reflected by the 0.5 m disk).

Panel B shows SPLs as a function of time for two of the waveforms in Panel A: left-reflector (left ear) and no-reflector (left ear). SPLs were calculated for running rectangular 1-ms windows with an overlap of 0.998 ms. The plots visualize the decay of the signal due to sound reflections in the room, the direct click reached a maximum of about 81 dB, and it took about 80 ms for the level to decline to the background level. The reflected click is seen in the left-reflector recording (solid black line), where it reached a maximum of about 87 dB at 10 ms (black arrow). The level of the no-reflector recording (dashed blue line) at the same time point was considerably lower, about 60 dB (blue arrow).

Panel C shows estimates of the time-interval between direct and reflected click (ICI, also known as echo-delay) with the Echobot in left-reflector position. The ICI increases with about 5.8 ms per meter distance to the reflecting disk, starting at about 5.2 ms at 1 m distance (the loudspeaker membrane generating the click was located about 0.7 m from the disk; therefore, the initial ICI was less than 5.8 ms). The small vertical distance between the data for the left (unfilled circles) and right ear (red squares) relates to the interaural time difference (about 0.15 ms, but it is difficult to derive reliable estimates of interaural time difference as these will depend on the exact positioning of the head, which will vary from measurement to measurement).

Panel D shows maximum 1-s SPLs of the reflected click as a function of distance to the reflector with the Echobot in left-reflector position. The vertical difference of about 9 dB between the data for the left (unfilled circles) and right ear (red squares) correspond to the interaural level difference (ILD). The level of reflected click was higher than the direct click (horizontal line) up to about 2 m distance to the disk for the ear closest to the disk (left ear). The maximum level of the overall stimulus (direct + reflected click) is equal to the maximum level of the stronger of the direct and the reflected click (about 89 dB at the closest distance, declining to 81 dB at about 2 m distance and further). Note that stimuli with the Echobot in no-reflector position would have a maximum level of about 81 dB at all distances, that is, equal to the maximum level of the direct click.

Panel E shows estimated ICIs with the Echobot in right-reflector position. The distance-ICI relationship was very similar to the one for left-reflector position (Panel C).

Panel F shows maximum 1-s SPLs with the Echobot in right-reflector position. The estimated ILD was 10 dB, similar to the left-reflector position. The points at which the reflected click was weaker than the direct click was about 2.3 m for the ear closest to the disk (right ear). The maximum level of the overall stimulus (direct + reflected click) ranged from about 90 dB at the closest distance, to about 81 dB from 2.3 m distance and further.

### Statistical Analysis

We summarize the results from the echolocation experiment with the sensitivity index *d'* and the response-bias index *c* from the standard normal and equal variance version of signal detection theory (Macmillan & Creelman, 2004). For the localization experiment, this involved arbitrarily defining one of the sides (left) as signal and the other (right) as noise to calculate hits and false alarms. In addition to point estimates, we also derived intervals around the estimates. We are following the recommendations of Amrhein et al. (2019) and use the term *compatibility interval* (CI) for these intervals instead of the less neutral term *credible interval* or *confidence interval*. Regardless of its name, the interval presents a range of values that are reasonably compatible with the data, given the assumptions of the statistical model used to compute it.

The conventional way to calculate *d ′* and *c* would be to calculate the proportions of hits (H) and false alarms (F) and then use the inverse normal cumulative distribution function (Φ^−1^) to calculate

(1) $$d^{\prime }=\Phi^{-1}\left(H\right)-\Phi^{-1}(F)$$

and

(2) $$c=-\frac{1}{2}\left[\Phi^{-1}(H)+\Phi^{-1}(F)\right]$$

There is no obvious way to calculate CIs around the estimates derived using Equations 1 and 2 (Suero et al., 2017), and estimates are undefined when *H* or *F* are equal to 1 or 0. An alternative method may therefore be preferred, namely to calculate *d'* and *c* from the raw data, $y_{i}$, without first calculating *H* and *F*. The following model may then be fitted to the data using a Bayesian estimation approach

(3) $$y_{i}\;∼\;\mathrm{Bernoulli}\left(p_{i}\right)$$

$$p_{i}=\left(1-S_{i}\right)\left[1-\Phi \left(c+\frac{d\prime }{2}\right)\right]+S_{i}\left[1-\Phi \left(c-\frac{d\prime }{2}\right)\right]$$

where $y_{i}$ is the response at trial *i*, coded 0 for *no, signal absent* and 1 for *yes, signal present,* modelled as an independent Bernoulli trial with probability $p_{i}$, Φ is the normal cumulative distribution function, $S_{i}$ is a dichotomous variable coded 1 if the signal was present at trial *i*, and coded 0 if not, and c and d' are parameters estimated from the data. These estimates will be the same as estimates derived using Equations 1 and 2 but with the advantages of avoiding issues with *H *=* *1.0 and *F *=* *0.0 and providing a straightforward way of calculating CIs. In the following, we will report the 95% highest posterior density interval (95% CI) together with the median (point estimate) of the marginal posterior distribution of model parameters or of measures derived from model parameters.

We analyzed the data individually by fitting two models to each participant’s data, using Bayesian inference and Hamiltonian Monte Carlo for estimating posterior distributions (e.g., Kuss et al., 2005). The first model, Equation 4, simply estimated *d'* and *c* separately for each of the 2 tasks and 10 distances to the Echobot’s disks (i.e., distance was treated as a categorical variable with 10 levels)

(4) $$y_{i}\;∼\;\mathrm{Bernoulli}\left(p_{i}\right)$$

$$p_{i}=\left(1-S_{i}\right)\left[1-\Phi \left(c_{T\left[i\right],\;D\left[i\right]}+\frac{{d^{\prime }}_{T\left[i\right],\;D\left[i\right]}}{2}\right)\right]+S_{i}\left[1-\Phi \left(c_{T\left[i\right],\;D\left[i\right]}-\frac{{d^{\prime }}_{T\left[i\right],Di]}}{2}\right)\right]$$

$${d^{\prime }}_{j},c_{j}\;∼\mathrm{Normal}\left(0,\;3\right)\;\mathrm{for}\;j=1.20$$

where *i* goes from 1 to 3,000 responses (150 per each of 2 tasks and 10 distances), $c_{T\left[i\right],\;D\left[i\right]}$ is the response criterion for task *i* at distance *i*, ${d'}_{T\left[i\right],\;D\left[i\right]}$ is the sensitivity index for task *i* at distance *i*; the same weakly informative prior was used for all 40 parameters of the model (normal distribution with mean = 0 and standard deviation = 3).

The second model, Equation 4, assumed an exponential relationship between *d ′* and distance (treated as a continuous variable)

(5) $$y_{i}\;∼\;\mathrm{Bernoulli}\left(p_{i}\right)$$

$$p_{i}=\left(1-S_{i}\right)\left[1-\;\Phi \left(c_{i}+\frac{{d^{\prime }}_{i}}{2}\right)\right]+S_{i}\left[1-\Phi \left(c_{i}-\frac{{d^{\prime }}_{i}}{2}\right)\right]$$

$${d^{\prime }}_{i}=\left(1-T_{i}\right)\alpha_{0}\exp\left(\alpha_{1}X_{i}\right)+T_{i}\beta_{0}\exp\left(\beta_{1}X_{i}\right)$$

$$c_{i}=\left(1-T_{i}\right)c_{0}+{T_{i}c}_{1}$$

$$\alpha_{0},\beta_{0},\;\alpha_{1},\beta_{1},c_{0},c_{1}∼\;\mathrm{Normal}\left(0,\;3\right),$$

where *i* goes from 1 to 3,000 responses, $T_{i}$ is a dichotomous variable coded 0 if the task at trial *i* was detection and 1 if it was localization, $X_{i}$ is the distance (m) to the Echobot’s disks at trial *i*, $\alpha_{0}$ and $\alpha_{1}$ are parameters of the exponential function relating *detection* d ′ to distance, $\beta_{0}$ and $\beta_{1}$ are parameters relating *localization* d' to distance, *c_0_* and *c_1_* are the response-bias parameters for the detection and the localization tasks, respectively (assumed to be the same for all distances), and the same weakly informative prior was used for all six parameters of the model.

All data and analysis scripts can be downloaded from Stockholm University (Nilsson et al., 2021; Tirado et al., 2021). 

## Results

The results suggested distinct individual differences in echo-detection and echo-localization abilities. This makes it less meaningful to aggregate data across individuals, and we will therefore present the results separately for each participant. The main results are presented in terms of the sensitivity index *d'*, estimated using two models. Model 1 (Equation 4) made no assumptions of the functional relationship between *d'* and distance, that is, estimates were derived separately for each of the 2 tasks × 10 distances (we treat these estimates as our “observed” data). Model 2 (Equation 5) assumed an exponential relationship between *d'* and distance, that is, it assumed a fixed proportional change in *d'* for each unit increase in distance. In the following figures, symbols with error bars refer to point estimates and 95% CIs derived from Model 1, whereas curves surrounded by shaded regions refer to estimated functions and 95% CIs derived from Model 2. In addition to *d'* estimates, Model 1 and 2 also provided estimates of the response-bias parameter *c*; these estimates are summarized in Table 1.

**Table 1.** Point Estimates of Response-Bias Index *c*, and Estimates of the Between-Task Difference in *c* With 95% Compatibility Interval.

	Model 1 (Equation 4)				Model 2 (Equation 5)			
	Echo-detection	Eco-localization	Difference		Echo-detection	Eco-localization	Difference	
ID	mean (c_0_)	mean (c_1_)	mean (c_0_–c_1_)	95% CI	γ_0_	γ_1_	γ_0_–γ_1_	95% CI
#1	–0.3	–0.1	–0.2	[–0.3, –0.1]	–0.2	–0.1	–0.1	[–0.2, 0.0]
#2	0.0	–0.1	0.1	[–0.1, 0.2]	0.1	–0.1	0.2	[0.1, 0.3]
#3	0.0	0.3	–0.3	[–0.5, –0.1]	0.2	0.3	–0.1	[–0.3, 0.0]
#4	0.0	–0.2	0.2	[0.0, 0.3]	0.2	–0.2	0.3	[0.2, 0.4]
#5	–0.2	0.3	–0.6	[–0.7, –0.5]	–0.2	0.3	–0.5	[–0.6, –0.4]
#6	0.1	0.2	–0.1	[–0.3, 0.0]	0.2	0.2	0.0	[–0.1, 0.1]
#7	–0.3	–0.1	–0.2	[–0.4, 0.0]	–0.2	0.0	–0.2	[–0.3, –0.1]
#8	0.2	0.2	0.0	[–0.2, 0.2]	0.4	0.2	0.2	[0.1, 0.3]
#9	–0.3	–0.1	–0.2	[–0.7, 0.2]	0.0	–0.2	0.1	[–0.1, 0.3]
#10	–0.1	0.3	–0.4	[–0.5, –0.3]	–0.1	0.3	–0.4	[–0.5, –0.3]

*Note*. c_0_ refers to response-bias parameters estimated from the echo-detection data, c_1_ is the corresponding estimate for the echo-localization experiment_._ For Model 1, mean(c_0_) and mean(c_1_) refer to mean values of 10 parameter estimates per task (*n* = 10 distances). Model 2 (Equation 5) had only one response-bias parameter per task. 95% CI = compatibility interval for the between-task difference in response-bias index.

**Figure 4.**
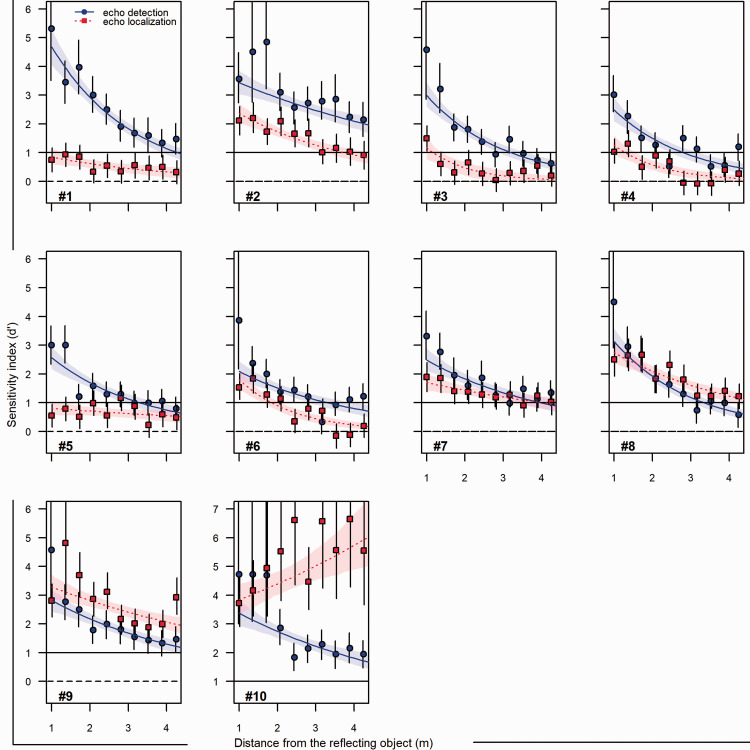
Individual performance in the echo-detection and the echo-localization task. Sensitivity index *d'* for echo-detection (blue circles and curves) and echo-localization (red squares and curves) as a function of distance to the reflecting disk, separately for each Participants #1, #2, …, #10. The horizontal line at *d ′* = 0 indicates chance performance (50% correct responses), and the line at *d* ′ 1 shows a performance better than chance corresponding to 69% correct responses for an unbiased responder. Error bars indicate the 95% compatibility interval (CI) around point estimates (Model 1, Equation 4). The curves and shaded regions show predictions from a model assuming a fixed proportional change in *d'* per unit increase in distance to the disk (Model 2, Equation 5). Note that the range of *y* axis values is the same in all panels, although scale values are different in the panel for Participant #10.

Figure 4 shows sensitivity index (*d'*) for echo-detection (blue circles) and echo-localization (red squares), separately for each participant, task, and distance to the disk. The horizontal dotted line in each panel at *d'* = 0 refer to chance performance (50% correct responses). The solid line at *d'* = 1 corresponds to 69% correct responses for an unbiased responder (*c *=* *0); we will consider *d'* > 1 as above-chance performance. The participant’s data have been ordered from the largest (#1) to the smallest (#10) average difference between detection *d'* and localization *d'*.

The general trend was that *d'* decreased with distance (Figure 4). For *echo-detection,* this was true for all participants, with clearly decreasing functions (blue curves) and only a few deviating observation (blue squares, e.g., Participant #2 at closer distances). The observed *d'* values agreed reasonably well with predicted values (Model 2, blue curves), consistent with the notion of a proportional decline in *d'* per unit increase in distance to the disks. A decreasing trend with distance was seen also for *echo-localization* (red squares and curves), although less distinct a trend for some participants (#1 and #5), and with the notable exception of Participant #10 who seemed to find it easier to localize the clicks at farther compared with closer distances.

For the closest distance to the disk (1 m), the observed *echo-detection d'* was greater than *echo-localization d'* for all participants (blue symbols located above red symbols in Figure 4). For seven of the participants (#1–#7), this was also true for further distances, up to 3 m or more if looking at the fitted functions (blue curves located above red curves). In contrast, Participants #8, #9, and #10 performed as well or better at localization than detection at distances > 1 m.

**Figure 5:**
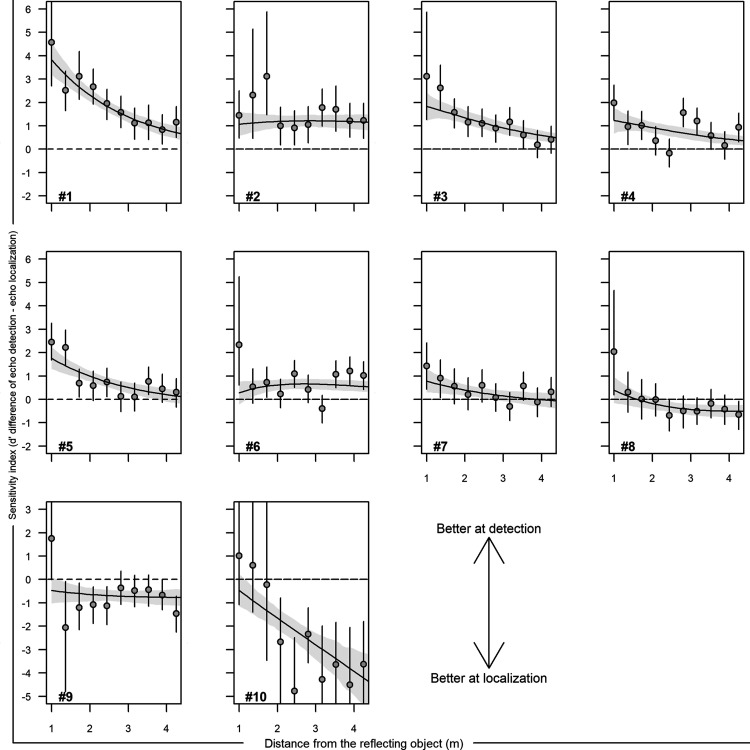
Individual *d ′* difference between tasks. Differences between echo-detection-*d'* and echo-localization *d'* as a function of distance to the reflecting disk, separately for each Participants #1, #2, …, #10. The horizontal line at *d ′* = 0 indicates no difference between the two tasks. Error bars indicate the 95% compatibility interval (CI) around differences between point estimates of *d ′* (Model 1, Equation 4). The curves and shaded regions show predicted differences from a model assuming a fixed proportional change in *d ′* per unit increase in distance to the disk (Model 2, Equation 5). Positive *d ′* differences means that the participant performed better at the echo-detection than the echo-localization task, and vice versa for negative *d'* differences. Note that the range of *y* axis values is the same in all panels, although scale values are different in the panels for Participants #9 and #10.

To compare directly the performance on the echo-detection versus echo-localization task, Figure 5 shows point estimates and 95% CIs for between-task differences in *d'* (data points in Figure 5 correspond to the vertical separation of circles and squares in Figure 4). A difference around zero would mean that performance was about the same in the two tasks, a difference substantially greater than zero implies better performance on the detection than the localization task, and vice versa for differences substantially lower than zero. Figure 5 adds to Figure 4 by giving CIs for the between-task differences in *d'* (not deducible from Figure 4). It also clearly illustrates the interaction between task and distance, suggesting a diminishing between-task difference with distance for six participants (#1, 3, 4, 5, 7, and 8), a roughly constant between-task difference for three participants (#2, 6, and 9), and an increasing between-task difference, in favor of localization, for Participant #10.

The primary focus of our analysis was the sensitivity index *d'*, our measure of echo-detection or echo-localization ability. However, it might also be of interest to look at the response-bias index *c*, to explore differences between the two tasks in their propensity to elicit predominantly *yes* (*c* < 0) or *no* (*c* > 1) responses. Model 1 estimated *c* separately for each of the 10 distances, yielding 10 response-bias estimates for each task. Mean values of these parameters and for the mean between-task difference are given in Table 1. Model 2 estimated *c* independently of distance, yielding one estimate per task, and these estimates are also given in Table 1. The table provides 95% CIs for the between-task comparisons of the response-bias parameter. The estimates in Table 1 are all close to zero, with small differences between tasks. Some participants seem to have adopted slightly different response criteria for the two tasks, most notably Participant #5 who had a liberal criterion in the detection task (*c *<* *0) and a conservative criterion in the localization task (*c *>* *0). However, overall, the estimates neither suggest that the two tasks provoked distinctly different response strategies, nor that any of our participants were strongly biased toward answering *yes* or *no.*

**Figure 6.**
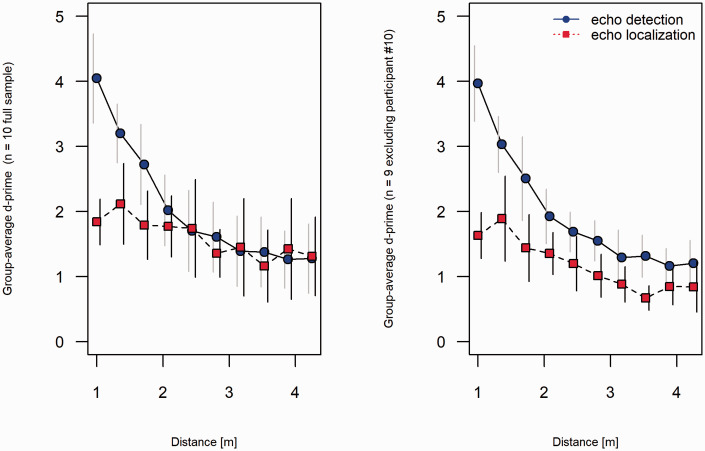
Group-averaged *d'* for echo-detection (circles) and echo-localization (localization) as a function of distance (m) to the reflecting disk. The horizontal dashed line at *d ′* = 1 shows a performance corresponding to approximately 69% correct responses for an unbiased responder. Error bars indicate 95% confidence intervals, derived using the method suggested by Cousineau & Paradis (2005).

Finally, as suggested by one anonymous reviewer, we also conducted group-level analysis using analysis of variance. We did this including all 10 participants as well as with Participant 10 excluded, as the data pattern of this participant clearly deviated from the pattern of the other participants (Figure 4). We used the 20 point estimates derived for each participant in the main analysis (Equation 1) as input in a 2 (Task) × 10 (Distance) repeated-measures analysis of variance. For the full sample of 10 participants, the F-ratios for the main effect of task were *F*(1, 9) = 1.29 (η^2^_partial_ = .13, *p *=* *.29), for the main effect of distance *F*(9, 81) = 33.45 (η^2^_partial_ = .79, *p *<* *.001), and for the Task × Distance interaction *F*(9, 81) = 9.28 (η^2^_partial_ = .51, *p *<* *.001). The corresponding group-averaged*d*′ values are shown in the left panel of Figure 6. For the restricted sample of 9 participants, the F-ratios for the main effect of task was *F*(1, 9) = 8.0 (η^2^_partial_ = .50, *p *=* *.02), for the main effect of distance *F*(9, 72) = 47.8 (η^2^_partial_ = .86, *p *<* *.001), and for the Task × Distance interaction *F*(9, 72) = 9.28 (η^2^_partial_ = .54, *p *<* *.001). The corresponding group-averaged *d*′ values are shown in the right panel of Figure 6. For detection, the two panels show similar trends. However, the exclusion of Participant 10, who performed extremely well on the localization task, make the group-averaged localization *d*′ values in the right panel consistently lower than the corresponding group-averaged detection-*d*′ values, whereas this was true only for the shortest distances in the full sample (left panel).

## Discussion

To our knowledge, this is the first study that compares the performance on an echo-detection and an echo-localization task in the same experiment and group of participants. Our results suggest distinct between-task differences, both within and across participants. A main result was that in general, and with notable exceptions, our participants performed better on the echo-detection than the echo-localization task. For short distances to the disk, several participants performed well on the echo-detection task, whereas their echo-localization performance was close to chance, illustrating that detectable sound reflections may be hard to localize. The practical implications of the results remain to be seen, but it may be relevant for echolocation training programs that could benefit from focusing separately on the detection and localization aspects of human echolocation.

The performance on the echo-detection was substantially better than on the echo-localization task for several of the participants, suggesting that they sometimes were unable to say whether an audible reflection came from the left or the right. It seems unlikely that this pattern of results was caused by differences in how hard it was to follow the task instructions. The tasks were very similar, both involving one stimulus and two response alternatives per trial. One difference was that the detection task involved three types of stimuli (left-reflector, no-reflector, and right-reflector), whereas the localization task only involved two of these (left-reflector and right-reflector) but, if anything, this would make the detection task slightly more difficult, not easier, than the localization task. The participants did not indicate that task instructions were harder to understand for one task to the other, and they were all given one full day of testing to get accustomed to the tasks before the main data collection started. Moreover, our analysis of the response-bias parameter *c* suggested that the two tasks evoked similar and fairly unbiased response patterns.

The acoustic analysis of the signals illustrates the complexity of the stimuli (Figure 3). This complexity points to several psychoacoustic phenomena that each may have contributed to the observed results, as discussed later.

First, at close distances, the reflected click had a higher maximum SPL than the direct click (see Panels D and F in Figure 3). Consequently, stimuli containing a reflected click would have a higher overall SPL than stimuli with no reflection, a difference of about 10 dB at the closets distance, diminishing up to about 2–2.5 m distance after which maximum levels would be similar for stimuli with and without reflections from the disk. In the *echo-detection* task, the main comparison was reflection present (left- or right-reflector) versus reflection absent (no-reflector). At close distance, reflection-present stimuli would have a higher maximum SPL (and thereby be louder) than reflection-absent stimuli. In contrast, the main comparison in the *echo-localization* task would be reflection to the left (left-reflector) versus reflection to the right (right-reflector). These stimuli would have about the same maximum level and thereby be similar in overall loudness, although their ILDs would point in opposite directions. Performance was better in the echo-detection task for most participants, especially at close distances, and this may suggest that it was easier to discriminate between differences in overall levels (echo-detection experiment) than between differences in the direction of ILDs (echo-localization experiment). Note that this was not obvious. The measured ILDs make sense given that the click was dominated by frequencies in the 3–4 kHz region, for which the head would be an effective screen of sounds reaching the farther ear. Previous research suggests that high-frequency sounds and large ILDs are particularly beneficial for echo-localization (Rowan et al., 2013, 2015; Schörnich et al., 2012), and 9 dB is a sizable ILD. Given this, it would not have been surprising if participants performed as well on the echo-localization task as on the echo-detection task.

Second, the time separation of direct and reflected clicks at the ear of the participant was about 5 ms at the closest distance and increasing with 5.8 ms per meter distance to the disk (Panels C and E of Figure 3). The signal had a duration of about 3 ms, which may suggest that the direct and reflected sounds were separated in time. However, this is not fully correct, as the reverberation time of the signal was about 80 ms, and the reflected sound occurred simultaneously with reverberations from the direct click (illustrated in Panel B of Figure 3). Thus, in addition to forward masking, simultaneous masking may have influenced the detectability of the reflected click. Masking may be complete, making the signal inaudible, or partial, lowering its loudness. It is possible that forward and simultaneous masking jointly reduced the loudness of the reflected click to the extent that it was hard to localize although it was still possible to detect.

Third, discrimination suppression refers to the loss of spatial information in an audible lag-click following a lead-click (Brown & Christopher Stecker, 2013). The present results have similarities with this phenomenon in that several participants found it hard to left-right localize stimuli that they could detect well above chance. According to the precedence-effect research, discrimination suppression tends to be largest for stimuli with an ICI of about 1–10 ms (Gaskell, 1983; Saberi & Perrott, 1990; Shinn‐Cunningham et al., 1993), although large individual differences typically are reported (Saberi et al., 2004; Saberi & Antonio, 2003). In our setup, the time between the direct sound and its reflection was <10 ms at our shortest distances (≤1.7 m), and it was for these distances that we observed the largest difference between echo-detection and echo-localization, consistent with findings from basic discrimination-suppression research. However, the acoustic analysis showed that the reflected click at these distances had a higher level than the direct click (Panel D and F, Figure 3). This may speak against discrimination suppression as an explanation of the poorer performance on the echo-localization task, as the loss of spatial information in lag-clicks would be less in scenarios where they are of greater amplitude than the lead-click (Pastore & Braasch, 2015). Again, the picture is complicated by the fact that our stimuli were not strictly separated in time (Panel B, Figure 3). One may speculate that interactions between the reflected click and reverberations of the direct click may have influenced access to spatial information, for example, at monaural stages of processing leading to conflicting ILDs within critical bands (cf. Nilsson et al., 2019; Xia & Shinn-Cunningham, 2011).

Fourth, yet another psychoacoustic phenomenon may be relevant, namely the faint pitch sensation (repetition pitch) evoked by the autocorrelation of combined correlated sounds (e.g., Bassett & Eastmond, 1964; Bilsen, 1966). The overlap in time between reverberations of the direct and reflected click in our study may have resulted in such physical autocorrelations that may have been detectable as repetition-pitch sensations, or, even if direct and reflect clicks were separated in time, a repetition-pitch sensation may result due to interactions in the auditory system between neural activities evoked by the clicks. Previous research has suggested that repetition pitch is a cue used for echo-detection (Schenkman & Nilsson, 2011), whereas it is unknown if this cue also is helpful for echo-localization.

The discussion earlier suggests several psychoacoustic phenomena that may be of relevance for our experimental results. Note, however, that our purpose was not to disentangle psychoacoustic mechanisms—that would have required another experiment, involving manipulation of experimental stimuli—but, as a first step, to explore the relationship between echo-detection and echo-localization.

In addition to comparing tasks within participants, it is relevant to compare tasks across individuals. All participants’ *echo-detection* sensitivity declined with distance in a way that was reasonably described by a simple model assuming a fixed proportional decrease in *d'* with each unit increase in distance (Model 2, Equation 5). Participants differed in the rate of the decrease, but they all seemed able to detect sound reflections better than chance (*d′* > 1) up to at least 3 m distance to the reflecting disk.

Compared with echo-detection, the echo-localization results showed less consistency across participants. Some participants were not able to localize better than chance at any but the closest distances, despite performing well on the echo-detection task (#1, #3, #4, #5 and #6 all had localization *d′* < 1 for most distances, see Figure 4). In contrast, one participant performed well (#9) and another exceptionally well (#10) on the echo-localization task. They were also the only participants who performed better (#9) or clearly better (#10) on the echo-localization than the echo-detection task. The two tasks were conducted on different days, and the tasks were not identical in terms of instructions and stimuli conditions (as discussed earlier). For these reasons, it would not be surprising to find small advantages for localization over detection in performance, at least for some participants (like Participant #9 and, for some distances, Participant #8). The exceptionally good performance of Participant #10 is, however, something different, and may suggest that this participant had discovered additional cues. For example, the participant may have picked up something in the combined sound of the disks moving and the masking noise, or have detected some faint reflection in the room that provided additional information on which side the disk was reflecting, or some other cue that was not available in the detection task. However, when asked after testing, Participant #10 reported that that the echo-localization task was easier and more intuitive but could not explain why. The true reason remain unclear to us, and we prefer to treat this result as an anomalous deviation from the general trend of similar or better performance on the echo-detection than echo-localization task. For future studies, the position of the reflecting objects could be manipulated to make the echo-localization task easier than the echo-detection task. We used 18-degree angles in this study, but it is likely that more extreme angles would facilitate echo-localization. Perhaps the performance differences between the two tasks are also related to the distance between the reflectors.

Strengths of our experiment include the use of real sound-reflecting objects, the large set of stimuli conditions (distances) presented in random orders, and the extensive testing of the participants over 4 days, one of which was devoted to training to get accustomed to the tasks. We used naïve participants and therefore used a prerecorded echolocation signal, not to confuse the ability to perceive sound reflections from the ability to produce efficient echolocation signals (cf. Tirado et al., 2019). This limits the generalizability of the results, as it is possible that expert echolocators using self-generated echolocation signals would show a different echo-detection versus echo-localization pattern. They would also be capable of adjusting their self-generated signal for different acoustics as needed to improve their echolocation performance. Our use of a single click and a passive listener situation also limits the ecological validity of the results, as an echolocator in real life typically is moving in space while repeatedly emitting echolocation signals. In addition, an echolocator may use head movements to facilitate echo-localization (Milne et al., 2014), whereas we restricted our participant’s head movements with a chin rest. These limitations notwithstanding, our results provide new data on the relationship between echo-detection and echo-localization of sound reflections in an echolocation scenario. We hope that our findings may inspire further research into task-specific and common mechanisms behind echo-detection and echo-localization.
